# Liraglutide, a glucagon-like peptide 1 receptor agonist, exerts analgesic, anti-inflammatory and anti-degradative actions in osteoarthritis

**DOI:** 10.1038/s41598-022-05323-7

**Published:** 2022-01-28

**Authors:** C. Meurot, C. Martin, L. Sudre, J. Breton, C. Bougault, R. Rattenbach, K. Bismuth, C. Jacques, F. Berenbaum

**Affiliations:** 1 4P-Pharma, Lille, France; 2 Sorbonne University, INSERM CRSA St-Antoine, Paris, France; 3 4Moving Biotech, Lille, France; 4 Sorbonne University, INSERM CRSA, Rheumatology Department, AP-HP St-Antoine, Paris, France

**Keywords:** Cell biology, Diseases, Rheumatology

## Abstract

Osteoarthritis (OA) is a common disabling disease worldwide, with no effective and safe disease-modifying drugs (DMOAD) in the market. However, studies suggest that drugs, such as liraglutide, which possess strong potential in decreasing low-grade systemic inflammation may be effective in treating OA. Therefore, the aim of this study was to examine the anti-inflammatory, analgesic, and anti-degradative effects in OA using in vitro and in vivo experiments. The results showed that intra-articular injection of liraglutide alleviated pain-related behavior in in vivo sodium monoiodoacetate OA mouse model, which was probably driven by the GLP-1R-mediated anti-inflammatory activity of liraglutide. Moreover, liraglutide treatment significantly decreased IL-6, PGE_2_ and nitric oxide secretion, and the expression of inflammatory genes in vitro in chondrocytes and macrophages in a dose-dependent manner. Additionally, liraglutide shifted polarized macrophage phenotype in vitro from the pro-inflammatory M1 phenotype to the M2 anti-inflammatory phenotype. Furthermore, liraglutide exerted anti-catabolic activity by significantly decreasing the activities of metalloproteinases and aggrecanases, a family of catabolic enzymes involved in cartilage breakdown in vitro. Overall, the findings of this study showed that liraglutide ameliorated OA-associated pain, possess anti-inflammatory and analgesic properties, and could constitute a novel therapeutic candidate for OA treatment.

## Introduction

Osteoarthritis (OA) is a prevalent disabling disease, affecting over 300 million individuals worldwide^1–3^. Owing to the pain and loss of function induced by this disease, it reduces the quality of life and causes a considerable economic burden estimated at $350 billion in the United States alone^4^. Additionally, because of the sedentary lifestyle associated with OA, it increases cardiovascular mortality by more than 50%, necessitating its listing in 2018 as a severe disease by the Food and Drug Administration (FDA)^5^. To date, there is no effective treatment for the disease, except for a few symptomatic drugs that are often weakly effective or poorly tolerated^6^.

OA is characterized by joint damage, affecting mainly the cartilage, synovial membrane, and subchondral bone. Synovitis, inflammation of the synovium, can occur in the early stages of OA^7,8^. OA synovitis directly contributes to several clinical signs and symptoms, including joint swelling and effusion, and reflects the structural progression of the disease^8,9^. Therefore, treatment capable of reducing synovial inflammation and slowing down cartilage destruction should have the essential characteristics of disease-modifying OA drugs (DMOADs).

In the early stage, inflammation leads to the secretion of inflammatory and pro-degradative mediators, including cytokines such as interleukin-1β (IL-1β), interleukin-6 (IL-6), and tumor necrosis factor-α (TNF-α), lipid mediators such as prostaglandin E2 (PGE_2_), and matrix metalloproteinases (MMPs) by macrophages and fibroblast-like synoviocytes^7,10^. These mediators can induce the production of MMPs and a disintegrin and metalloproteinase with thrombospondin motifs (ADAMTS) by chondrocytes and further degradation of the cartilage, contributing to a cycle of inflammation and disease^7,10^.

Moreover, macrophages are the main immune cell type in healthy synovium and are likely the front-line cells that sense joint damage. These cells also contribute to OA progression by producing MMPs and cytokines^11^. Studies have shown that macrophages accumulate and become polarized towards the M1 phenotype in the synovium during OA development^12^.

Among the explanations for joint inflammation observed during OA is the hypothesis that the low-grade systemic inflammation observed during metabolic diseases could be one of the factors initiating and aggravating the OA process^13^. Several mechanisms have been implicated in the pathophysiology of diabetes-induced OA. Chronic hyperglycemia results in the accumulation of advanced glycation end products (AGEs), which is higher in diabetic patients than in normal patients^14^. AGEs activate inflammatory and oxidative pathways within the joint tissue^15^. Furthermore, hyperglycemia exerts synergistic effects with IL-1β and can directly increase oxidative stress and inflammation. Thus, hyperglycemia may increase responsiveness to local low-grade inflammation^16,17^. We hypothesized that drugs that have previously demonstrated strong potential in decreasing low-grade systemic inflammation could also act locally in the joint. Glucagon-like peptide-1 (GLP-1) is an incretin, a hormone secreted by the gut during meals that triggers the pancreas to produce insulin, which possesses a spectrum of extra-pancreatic functions related to its anti-inflammatory properties^18–20^. Liraglutide, commercially known as Victoza^®^, is a modified human GLP-1(7–37) with a longer half-life and is administered for type II diabetes^21^. GLP-1 receptor (GLP-1R) is expressed in pancreatic islets as well as in several extra-pancreatic organs or cell lineages, indicating that GLP-1-based drugs can exert extra-pancreatic functions. GLP-1 shows anti-inflammatory properties in pancreatic islets and adipose tissue, contributing to lower glucose levels in diabetic patients^22,23^. In addition to these tissues, emerging data suggest that GLP-1-based therapies possess anti-inflammatory effects on the liver, brain, kidney, lung, testis, skin, and vascular system including aorta and vein endothelial cells, by reducing the production of inflammatory cytokines and infiltration of immune cells in the tissues^19,24^. Thus, we speculated that GLP-1-based therapies, especially liraglutide^25^, may be beneficial for the treatment of OA due to its anti-inflammatory and anti-catabolic effects as well as its potential analgesic properties when injected intra-articularly. However, the anti-inflammatory mechanism of liraglutide is poorly understood.

Therefore, the aim of this study was to examine the analgesic and anti-inflammatory properties of liraglutide in treating OA, using in vivo and in vitro experiments. The findings of this study could provide potential therapeutic materials or target for treating OA. Here, we show here that GLP-1R is expressed in the cartilage and synovium in human diseased and mouse normal joint tissues. Moreover, we demonstrated that intra-articular (IA) liraglutide alleviates pain-related behavior in a sodium monoiodoacetate (MIA) OA mouse model displaying an analgesic effect. This analgesic effect observed in vivo is likely underlain by liraglutide’s concerted anti-inflammatory action, as demonstrated in vivo in a mouse model of MIA, whereby liraglutide improves synovitis severity score, and also in vitro*,* whereby liraglutide dose-dependently decreased PGE_2,_ nitric oxide (NO), and IL-6 secretion, along with *iNos*, *Cox2*, and *Tnf-α* gene expression levels in both chondrocytes and macrophages. In addition, liraglutide is able in vitro to shift the polarized macrophage phenotype from the pro-inflammatory M1 phenotype to the M2 anti-inflammatory phenotype. The ability of the GLP-1R antagonist, exendin 9–39, to compete with away liraglutide’s anti-inflammatory effects, confirmed that GLP-1R is the primary target of liraglutide in chondrocytes and macrophages. Finally, our in vitro results indicated that liraglutide may attenuate cartilage degradation through an anti-catabolic effect at both at the protein and gene expression levels. Targeting inflammation and cartilage breakdown in two main cellular actors of the diseased joint, and alleviating pain in vivo, confers to liraglutide the properties of a potential disease modifier, which could constitute a new treatment for OA.

## Results

### GLP-1 receptor is expressed in articular cartilage and synovial membrane in human and mice

**Figure 1:**
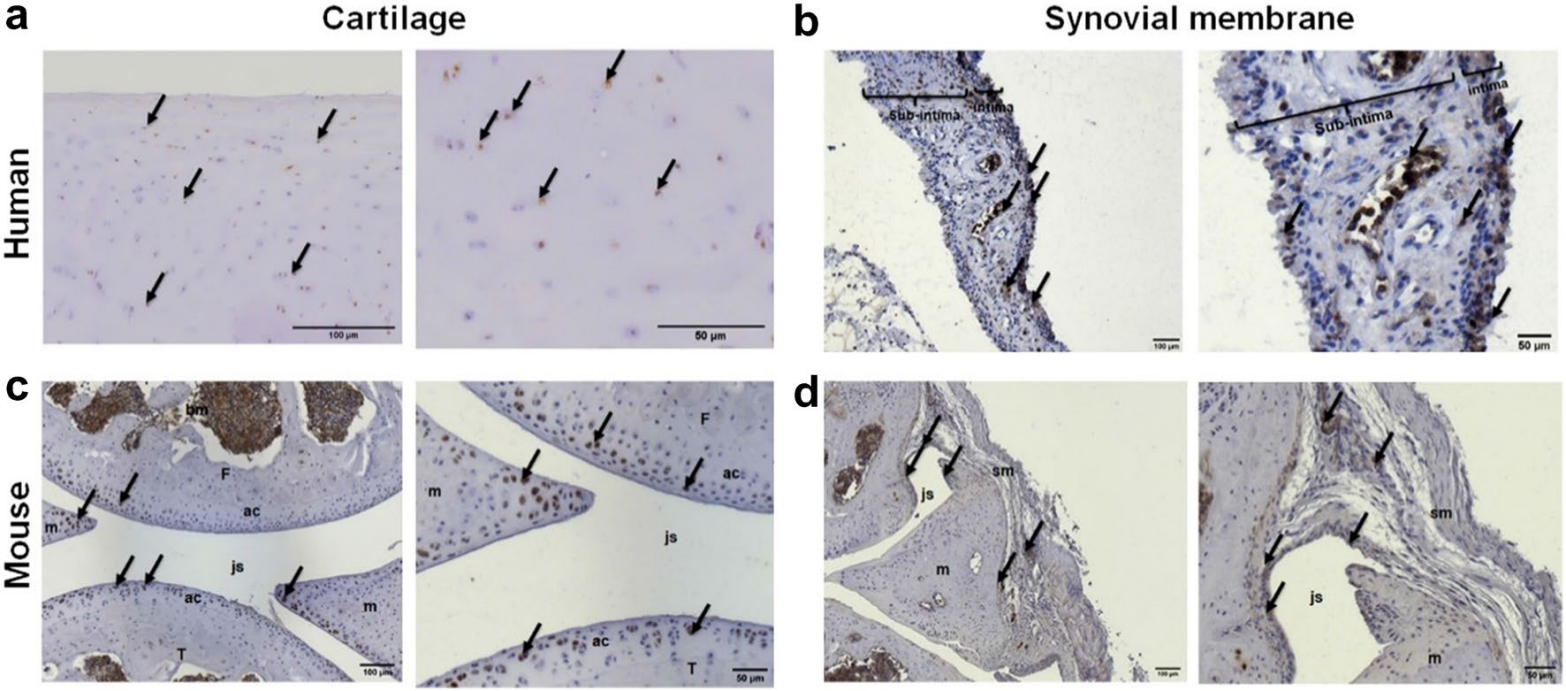
Expression of GLP-1 receptor in OA human and non-OA mouse knee joint. (**a**) Immunohistochemical staining of human OA knee cartilage sections was performed to determine the presence of GLP-1R (Mankin score: 3/14, scale bar = 100 µm or 50 µm). (**b**) Immunohistochemical staining of human OA synovial membrane sections was performed to determine the presence of GLP-1R (*si:* subintima, *i:* intima, *v:* blood vessel, scale bar = 100 µm or 50 µm). (**c**) Immunohistochemical staining of non-OA mice knee joint sagittal sections to determine the presence of GLP-1R (*ac:* articular cartilage, *m:* meniscus, *js:* joint space, *T:* tibia, *F:* femur, *bm:* bone marrow, scale bar = 100 µm or 50 µm). (**d**) Immunohistochemical staining of non-OA mice synovial membrane sagittal sections to determine the presence of GLP-1R (*sm:* synovial membrane, *js:* joint section, *m:* meniscus, scale bar = 100 µm or 50 µm). Control experiment was performed without primary antibody incubation. Arrows indicate example of cells positive for GLP-1R staining.

Immunohistochemical examination of superficial and intermediate layer cartilage sections of human OA knee joints indicated that chondrocytes express the GLP-1R protein (Fig. 1a). Additionally, positive GLP-1R staining was observed in human synovial membrane sections from OA patients, specifically at the intima and blood vessel levels (Fig. 1b). Furthermore, immunohistochemical analysis of the knee joints of mouse indicated positive GLP-1R staining in the tibia and femoral articular cartilage, meniscus, and bone marrow of healthy murine knee articular sections (Fig. 1c). Similarly, a cluster of synovial cells showed positive staining for GLP-1R in healthy murine synovial membrane (Fig. 1d). Overall, these results suggest a central and pleiotropic potential role of GLP-1R in normal and diseased knee joints from human and mouse species. Expression of GLP1-R was confirmed by RT-qPCR analysis of RNA from primary human chondrocytes from cartilage explants from 18 human OA patients (data not shown).

### Liraglutide exerts analgesic and anti-inflammatory effects in vivo in a short-term sodium monoiodoacetate murine model of osteoarthritis

**Figure 2:**
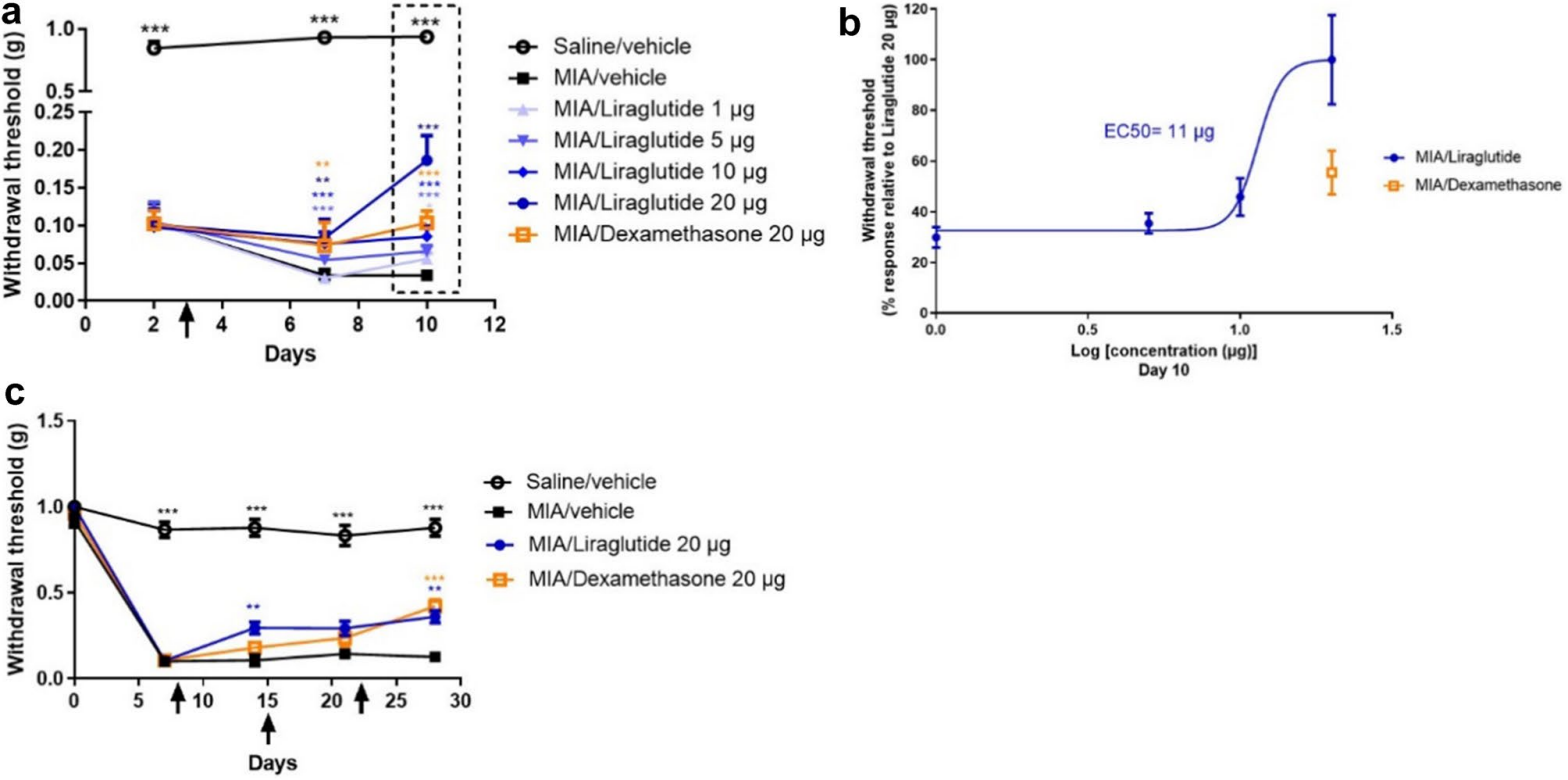
Liraglutide displayed analgesic effect in MIA mice models of OA. Mice knee joints were intra-articularly (IA) injected with 0.75 mg of MIA or saline on day 1. For the short-term study (**a,b**), treatments (liraglutide, dexamethasone, or vehicle) were injected IA on day 3 and inflammation pain sensitivity was determined by the von Frey test on day 2 (for randomization), 7, and 10 (n = 15–19 per group). For the long-term study (**c**), treatments were administered on days 8, 15, and 22, and von Frey tests were performed on day 7 (for randomization), 14, 21, and 28 (n = 9–10 per group). (**a**) Paw withdrawal threshold was assessed by von Frey filament stimulation on days 2, 7, and 10. (**b**) The efficacy rate of liraglutide in the MIA short-term study was analyzed using GraphPad Prism 9.0 and the EC_50_ value was determined at day 10 from the von Frey calculated values. (**c**) Paw withdrawal threshold was assessed by von Frey filament stimulation on days 0, 7, 14, 21, and 28. Arrows indicate the treatment with IA administration. Statistical analysis: Mean ± SEM. Mann–Whitney test with sequential strategy, **p < 0.01, ***p < 0.001, ****p < 0.0001 versus MIA control.

**Figure 3:**
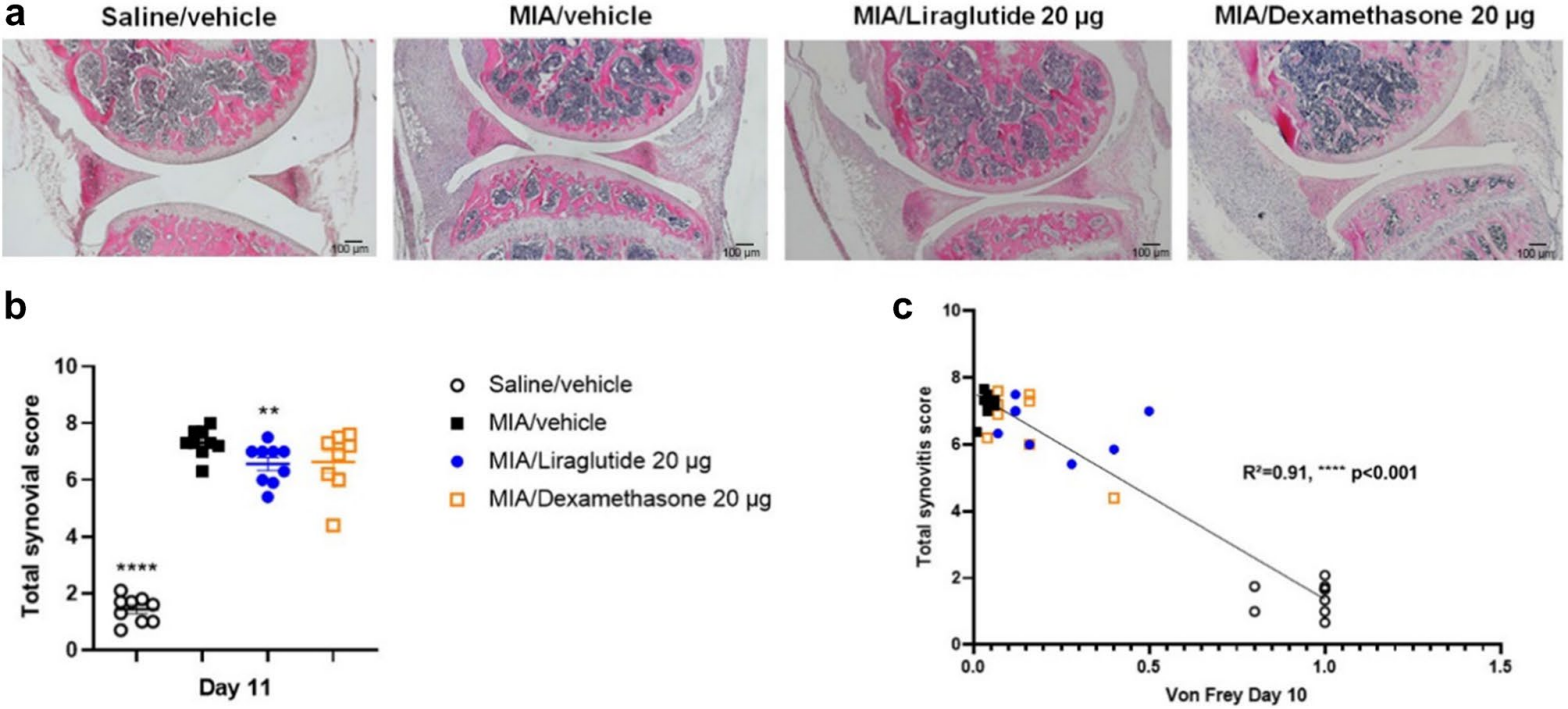
Liraglutide displayed anti-inflammatory effect in vivo in short-term MIA mice model of OA. Mice knee joints were intra-articularly (IA) injected with 0.75 mg of MIA or saline on day 1. Treatments (liraglutide, dexamethasone, or vehicle) were IA injected on day 3 (n = 15–19 per group). At the end of the study, on day 11, the mice were euthanized, and the right knee joint was recovered for histological analyses. (**a**) Representative photographs of sagittal sections of the paws of mice IA injected with 0.75 mg of MIA to induce inflammation and subsequently IA treated with vehicle, liraglutide or dexamethasone (positive control). (**b**) Histogram representing the total synovitis score calculated from Krenn et al*.*, synovitis score system (n = 8–9 per group). (**c**) Based on the results of the von Frey test on day 10 and the synovitis score obtained for each animal, a correlation curve between these two parameters was calculated using GraphPad Prism 9.0 (n = 8–9 per group). Statistical analysis: Mean ± SEM. Mann–Whitney test with sequential strategy, **p < 0.01, ****p < 0.0001 versus MIA control. Simple linear regression, ****p < 0.0001.

The effect of intra-articular injection of liraglutide was evaluated in vivo using short-term (up to 10 days) MIA OA mice model. The effect of liraglutide on OA-associated pain was assessed using the von Frey test. In this model, behavioral experiments were performed on day 2 (randomization), 7 and 10. Following IA injection of MIA, mice exhibited a significant decrease in paw withdrawal threshold as early as day 2 (*p* < 0.0001) compared to saline-treated mice and the difference in mechanical allodynia remained statistically significant until the end of the experiment (Fig. 2a). On day 3, that is 2 days after MIA model induction, a single IA injection of liraglutide induced a significant dose-dependent increase in paw withdrawal threshold compared with the vehicle-treated group on day 7 (liraglutide 5 µg *p* < 0.0001, liraglutide 10 µg *p* = 0.0002, and liraglutide 20 µg *p* = 0.0011), with 20 µg of liraglutide having similar effects with 20 µg of dexamethasone (positive control, *p* = 0.0022). Similarly, there was a significant dose-dependent increase in paw withdrawal threshold in the liraglutide-treated groups compared with the vehicle group on day 10 (liraglutide 1 µg *p* = 0.0196, liraglutide 5 µg *p* = 0.0004, and liraglutide 10 µg and 20 µg *p* < 0.0001, compared with the vehicle-treated group). Notably, the efficacy of liraglutide 20 µg was superior to that of dexamethasone (*p* < 0.0001) at day 10. The calculated median effective concentration (EC_50_) was 11 µg (Fig. 2b). Based on the results of the short-term model, 20 µg liraglutide was selected for histopathological analyses (Fig. 3). Liraglutide treatment significantly improved synovitis severity score in the MIA mice model (saline/vehicle *p* < 0.0001, liraglutide 20 µg *p* = 0.0099, compared to MIA/vehicle-treated group) (Fig. 3a,b). Although dexamethasone 20 µg also reduced the total Krenn score^26^, its effect was not significant (dexamethasone 20 µg *p* = 0.1288) (Fig. 3b). Furthermore, correlation analysis between synovitis score (Fig. 3b) and the results of the von Frey test at day 10 (Fig. 2a,b) indicated 91% correlation (R^2^ = 0.91 *p* < 0.0001) between inflammation and pain. Overall, liraglutide treatment successfully improved the synovitis severity score in the short-term MIA mice model. The anti-pain effect observed in this study could be, at least partially, mediated by the anti-inflammatory effect of liraglutide.

### Liraglutide exerts analgesic effect in vivo in a long-term sodium monoiodoacetate murine model of osteoarthritis

The effect of intra-articular injections of liraglutide were evaluated in vivo using a long-term (up to 28 days) MIA mice model. Based on the results of the short-term model, 20 µg liraglutide was selected, and the potential benefit of liraglutide in OA-associated pain was assessed using the von Frey test (Fig. 2c). Behavioral experiments in repeatedly treated animals were performed on day 7 (for randomization), 14, 21, and 28. MIA injection significantly reduced the withdrawal threshold of the ipsilateral paw compared with the saline group until the end of the study. Liraglutide induced a significant improvement in mechanical allodynia over time compared with the vehicle-treated group (day 14: liraglutide 20 µg *p* = 0.0098, day 28: liraglutide 20 µg *p* = 0.0038, dexamethasone 20 µg *p* = 0.0002, compared to vehicle-treated group).

Similar to the short-term model, 20 µg of liraglutide was more efficient than dexamethasone on day 14 (liraglutide 20 µg *p* = 0.0098, dexamethasone 20 µg *p* = 0.0568). After the second and third injections, the effects of dexamethasone and liraglutide were comparable, with comparable analgesic effects at day 21 and day 28. This clearly suggests that liraglutide acts faster than dexamethasone in relieving pain symptoms in this model.

Additionally, the treatments did not significantly affect the body weight of the animals in both the short- and long-term studies (Suppl. Fig. 1).

Taken together, these results indicated that intra-articular injection of liraglutide possess rapid analgesic effects compared to the standard treatment, and that local administration of liraglutide was well tolerated.

Furthermore, we hypothesized that the analgesic effect of liraglutide is dependent, at least partially, on its anti-inflammatory properties. To verify this assumption, an in vitro experiment was conducted using two murine cell types (chondrocytes and macrophages), which reflect two crucial cell types present in knee joints. Immunohistochemical assay indicated positive GLP-1R staining at the membrane and intracellular levels in both chondrocytes and RAW 264.7 murine macrophage cell lines by immunofluorescence (Suppl. Fig. 2). Therefore, these two cell models were used to examine the potential anti-inflammatory effect of liraglutide.

### Liraglutide exerts dose-dependent anti-inflammatory effect on murine primary chondrocytes

**Figure 4:**
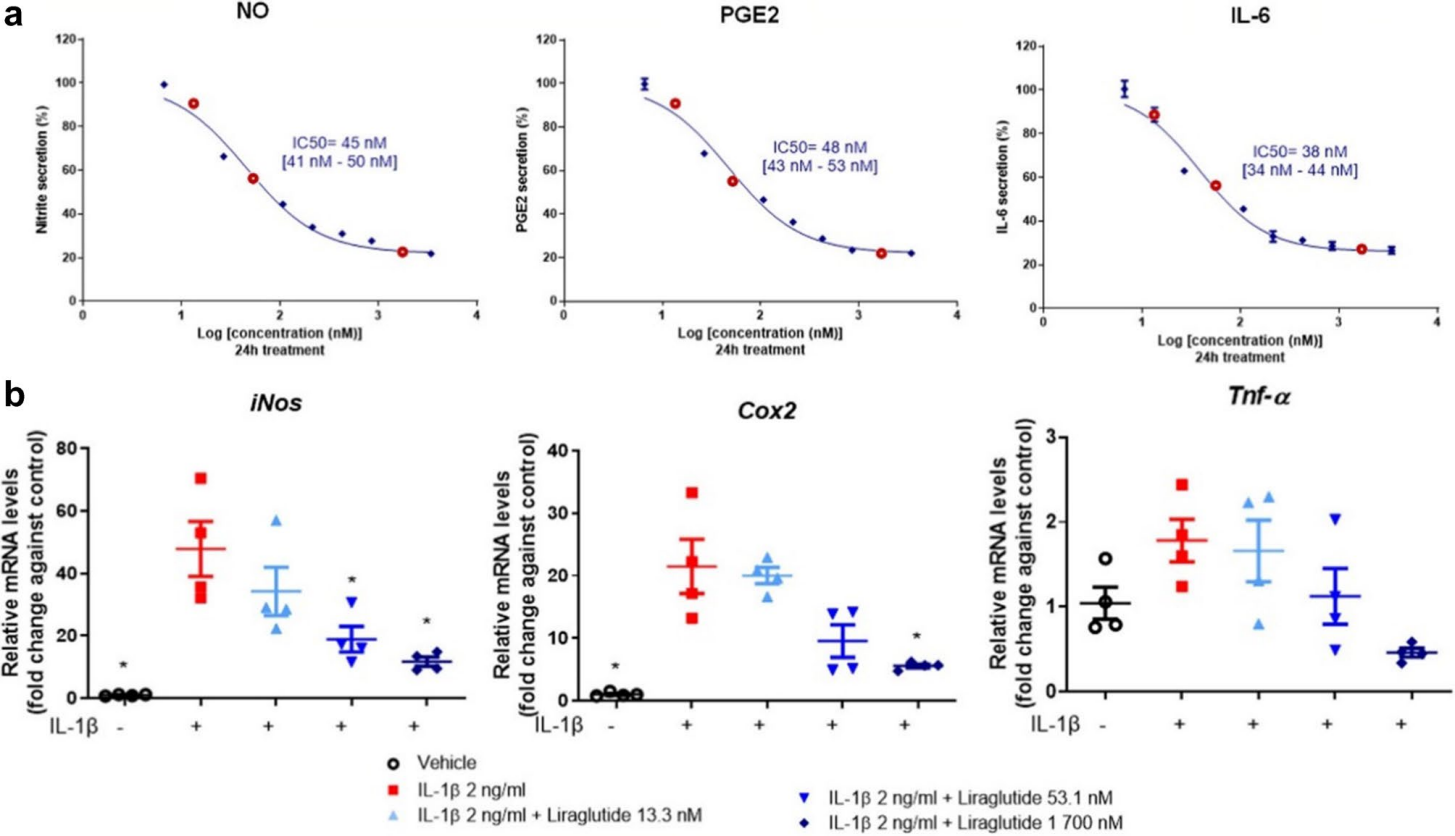
Anti-inflammatory effects of liraglutide in murine primary chondrocytes. Murine primary chondrocytes were stimulated with IL-1β (2 ng/mL) and co-treated with 10 doses of liraglutide (6.6, 13.3, 26.6, 53.1, 106.3, 212.5, 425, 850 nM, 1.7 and 3.4 µM) for 24 h (n = 4). (**a**) The inhibition rate of liraglutide in murine primary chondrocytes was analyzed using GraphPad Prism 9.0, and the IC_50_ values were determined for NO, PGE_2_, and IL-6, which were detected in the culture supernatant. (**b**) Relative mRNA expression of *iNos*, *Cox2*, and *Tnf-α* in murine primary chondrocytes co-treated for 24 h with 2 ng/mL IL-1β and liraglutide (13.3 nM, 53.1 nM, and 1 700 nM, shown in red circles in (**a**)) or vehicle (n = 4). Statistical analysis: Mean ± SEM, Mann–Whitney test with sequential strategy, *p < 0.05, versus stimulated control (IL-1β alone).

To investigate the in vitro effect of liraglutide on the production of inflammatory mediators in chondrocytes, cells were cultured with 2 ng/mL IL-1β for 24 h. IL-1β-induced chondrocytes were incubated with 10 ascending doses (6.6, 13.3, 26.6, 53.1, 106.3, 212.5, 425, 850 nM, 1.7 and 3.4 µM) of liraglutide for 24 h to determine the half maximal inhibitory concentration (IC_50_) of the inflammatory mediators NO, PGE_2_, and IL-6 (Fig. 4a). Three doses were selected for gene expression analyses (13.3 nM, 53.1 nM, and 1700 nM, shown in red circles in Fig. 4a). Lactate dehydrogenase assay (LDH) was performed to confirm the non-cytotoxicity of liraglutide treatment on primary murine chondrocytes (data not shown). The results showed that IL-1β stimulation induced an increase in the secretion of the three pro-inflammatory mediators. Remarkably, liraglutide treatment significantly reduced IL-1β-induced secretion of nitrite, PGE_2_, and IL-6 in a dose-dependent manner (Fig. 4a). The calculated IC_50_ was 45 nM with confidence interval [41–50 nM] for nitrite secretion, 48 nM [43–53 nM] for PGE_2_ secretion, and 38 nM [34–44 nM] for IL-6 secretion (Fig. 4a). Similarly, liraglutide treatment significantly decreased IL-1β-induced expression of *iNos, Cox2* and *Tnf-α* in chondrocytes (*p* = 0.0286 compared to IL-1β group) (Fig. 4b, Supplemental Table 1). Overall, these results demonstrated that liraglutide induced broad anti-inflammatory dose-dependent effects in IL-1β-stimulated murine chondrocytes.

### Liraglutide exerts dose-dependent anti-inflammatory effect on murine macrophages

**Figure 5:**
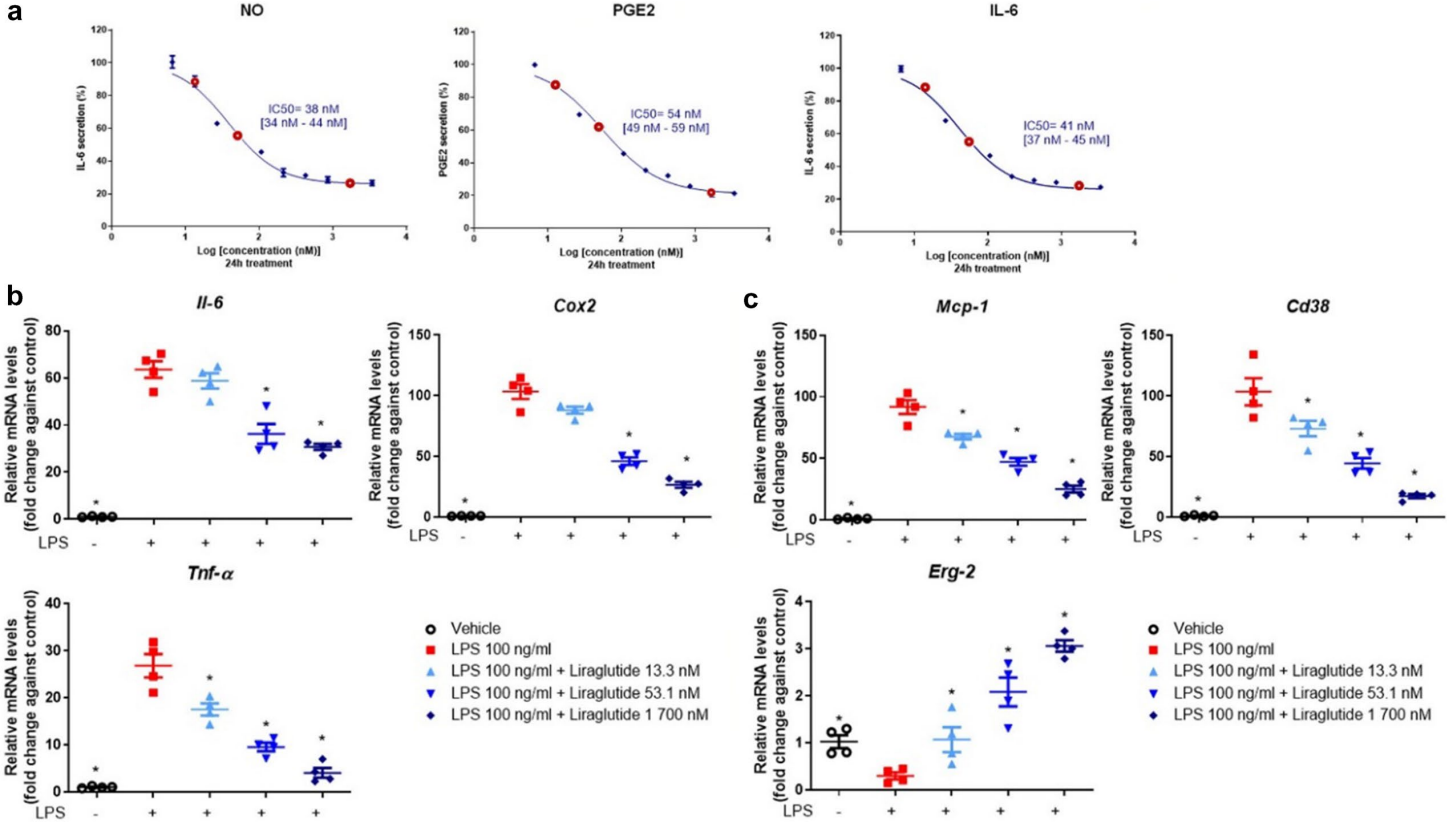
Anti-inflammatory effects of liraglutide in murine macrophages. Murine macrophage RAW 264.7 cells were stimulated with 100 ng/mL of LPS and co-treated with 10 doses of liraglutide (6.6, 13.3, 26.6, 53.1, 106.3, 212.5, 425, 850 nM, 1.7 and 3.4 µM) for 24 h (n = 4). (**a**) The inhibition rate of liraglutide on RAW 264.7 cell line was analyzed by GraphPad Prism 9.0, and the IC_50_ values were determined for NO, PGE_2_, and IL-6 detected in the culture supernatant. (**b**) Relative mRNA expression of *Il-6*, *Cox2*, *Tnf-α* in RAW 264.7 cell line co-treated with LPS and liraglutide (13.3 nM, 53.1 nM, and 1 700 nM, shown in red circle in graphs (**a**)) or vehicle for 24 h (n = 4). (**c**) Relative mRNA expression of *Mcp-1*, *Cd38*, and *Erg-2* in RAW 264.7 cell line treated with LPS and liraglutide (13.3 nM, 53.1 nM, and 1 700 nM, shown in red circle in (**a**)) or vehicle for 24 h (n = 4). Statistical analysis: Mean ± SEM, Mann–Whitney test with sequential strategy, *p < 0.05, versus stimulated control (LPS alone).

Considering the central role of macrophages in OA and the expression of GLP-1R in the macrophage cell line (Suppl. Fig. 2), the anti-inflammatory effect of liraglutide on RAW 264.7 cell line was examined. RAW 264.7 cells were cultured with 100 ng/mL of LPS at 10 ascending doses (6.6, 13.3, 26.6, 53.1, 106.3, 212.5, 425, 850 nM, 1.7 and 3.4 µM) of liraglutide for 24 h to determine the IC_50_ of the pro-inflammatory mediators NO, PGE_2_, and IL-6 (Fig. 5a). Lactate dehydrogenase assay (LDH) was performed to confirm the non-cytotoxicity of liraglutide treatment in a murine macrophage cell line (data not shown). The results indicated that liraglutide treatment significantly reduced LPS-induced secretion of NO, PGE_2_, and IL-6 in RAW 264.7 cells in a concentration-dependent manner (Fig. 5a). The calculated IC_50_ was 38 nM with confidence interval [34–44 nM] for nitrite secretion, 54 nM [49–59 nM] for PGE_2_ secretion, and 41 nM [37–45 nM] for IL-6 secretion (Fig. 5a). Similarly, liraglutide treatment significantly reduced LPS-induced upregulation of *Il-6*, *Cox2*, and *Tnf-α* in RAW 264.7 cells (*p* = 0.0286 compared to LPS group) in a dose-dependent manner (Fig. 5b, Supplemental Table 1).

Macrophage polarization is subject to environmental cues. To test whether liraglutide can reverse the inflammatory phenotype of macrophages, LPS-stimulated RAW 264.7 cells were exposed to three increasing doses of liraglutide. Results showed that there was a downregulation in the expression of M1 pro-inflammatory macrophage-related genes, including *Mcp-1* and *Cd38* (Fig. 5c). Conversely, there was an upregulation in the expression of *Erg-2*, a M2 phenotype-associated gene. These results indicated that liraglutide promoted macrophage repolarization from the pro-inflammatory M1 to the anti-inflammatory M2 phenotype in vitro, and that liraglutide could prevent or resolve inflammation at the tissue level.

### The anti-inflammatory effect of liraglutide is mediated by the GLP-1 receptor signaling pathway

**Figure 6:**
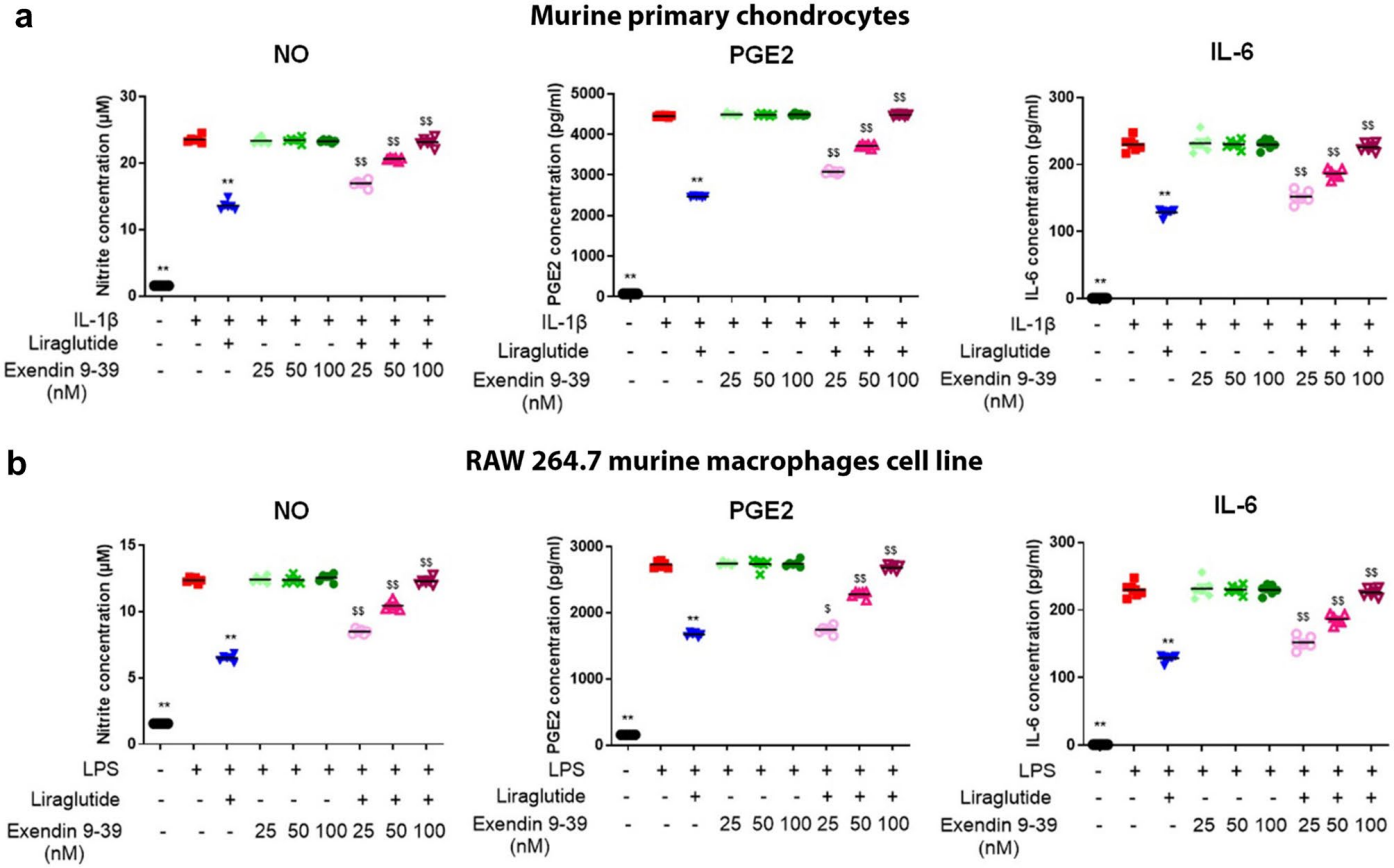
Anti-inflammatory effects of liraglutide are mediated by the GLP-1 receptor pathway. Primary cultured murine articular chondrocytes were incubated with 2 ng/mL IL-1β (**a**) and RAW 264.7, which were incubated with 100 ng/mL LPS (**b**) and co-treated with 50 nM of liraglutide or co-treated with three doses of exendin fragment 9–39 (25 nM, 50 nM, and 100 nM) for 24 h (n = 6). Nitrite, PGE_2_, and IL-6 concentration of the supernatant was determined. Statistical analysis: Mean ± SEM, Mann–Whitney test with sequential strategy, **p < 0.01, versus stimulated control (IL-1β or LPS) and ^$^*p* < 0.05, ^$$^*p* < 0.01, versus IL-1β + liraglutide or LPS + liraglutide group.

To determine whether GLP-1R was the sole target and mediator of the anti-inflammatory effects of liraglutide, we performed a functional competition assay using exendin 9–39, a GLP-1R competitive antagonist, in both chondrocytes and macrophages (Fig. 6). The chondrocytes and macrophages were exposed to liraglutide and increasing doses of exendin 9–39. The results showed that exendin 9–39 restored nitrite, PGE_2_, and IL-6 secretion in liraglutide pretreated chondrocytes and macrophages in a dose-dependent manner (*p* = 0.0022 compared with IL-1β + liraglutide group and *p* = 0.026 or *p* = 0.0022 compared with LPS + liraglutide group). Treatment with 100 nM of exendin 9–39 completely reversed the anti-inflammatory effect obtained with 50 nM (IC_50_ value) liraglutide. These data suggest that GLP-1R is the primary target of liraglutide in both chondrocytes and macrophages and that the anti-inflammatory effect of liraglutide is mediated by the GLP-1 receptor in these cell types (Fig. 6a,b).

### Liraglutide exerts anti-catabolic effect in murine primary chondrocytes in vitro

**Figure 7:**
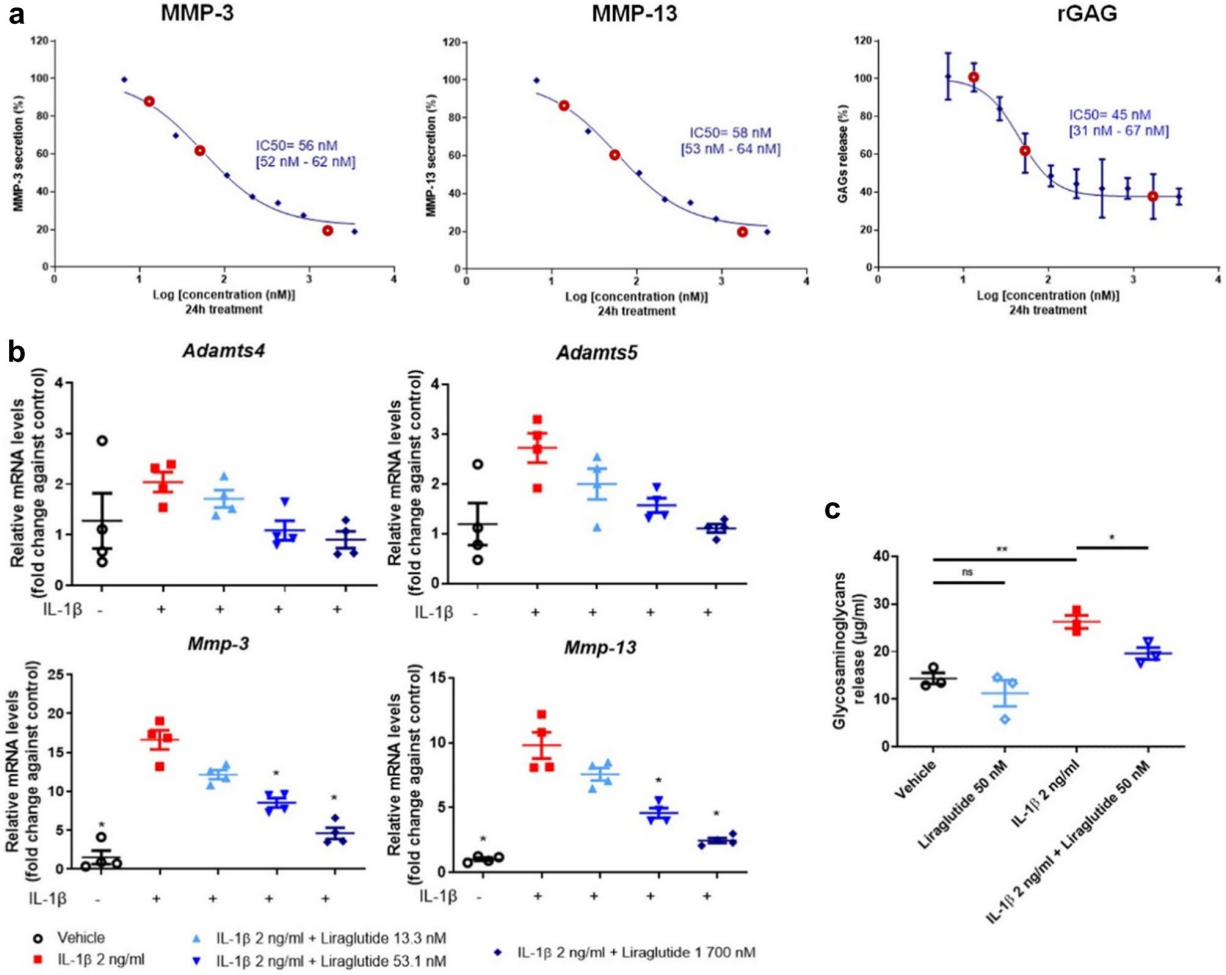
Anti-catabolic effects of liraglutide on murine primary chondrocytes. Murine primary chondrocytes were co-treated with 2 ng/mL of IL-1β and 10 doses of liraglutide (6.6, 13.3, 26.6, 53.1, 106.3, 212.5, 425, 850 nM, 1.7 and 3.4 µM) for 24 h (n = 4). (**a**) The inhibition rate of liraglutide in murine primary chondrocytes was analyzed using GraphPad Prism 9.0 and the IC_50_ values were determined. The expression of MMP-3, MMP-13, and GAG (rGAG) was detected in the cell supernatant. (**b**) Relative mRNA expression of *Adamts4*, *Adamts5*, *Mmp-3*, and *Mmp-13* in murine primary chondrocytes co-treated with IL-1β and liraglutide (13.3 nM, 53.1 nM, and 1 700 nM) or vehicle for 24 h (n = 4). (**c**) Murine primary chondrocytes were stimulated with 2 ng/mL of IL-1β and co-treated with 50 nM of liraglutide for 72 h (n = 3). The concentration of glycosaminoglycans was determined in the culture supernatant using the GAG assay. Statistical analysis: Mean ± SEM, Mann–Whitney test with sequential strategy, *p < 0.05, **p < 0.01, versus stimulated control (IL-1β alone). *ns* non-statistical.

To explore the inhibitory effect of liraglutide on the production of catabolic mediators, IL-1β-stimulated chondrocytes were cultured with 2 ng/mL of IL-1β and 10 ascending doses (6.6, 13.3, 26.6, 53.1, 106.3, 212.5, 425, 850 nM, 1.7 and 3.4 µM) of liraglutide for 24 h for IC_50_ determination. The calculated IC_50_ was 56 nM with confidence interval [52–62 nM] for MMP-3 secretion, 58 nM [53–64 nM] for MMP-13 secretion, and 45 nM [31–67 nM] for GAG secreted from extracellular matrix (Fig. 7a). Moreover, the effect of three selected doses (13.3 nM, 53.1 nM, and 1700 nM) of liraglutide on *Adamts4*, *Adamts5*, *Mmp-3*, and *Mmp-13* expression in IL-1β-stimulated chondrocytes was evaluated. Results showed that liraglutide treatment significantly reduced (*p* = 0.0286) IL-1β-induced expression of *Adamts4*, *Adamts5*, *Mmp-3*, and *Mmp-13* in a dose-dependent manner compared with the IL-1β group (Fig. 7b). Furthermore, to explore the long-term anti-degradative effect of liraglutide, IL-1β-stimulated chondrocytes were cultured with 2 ng/mL of IL-1β and 50 nM of liraglutide for 72 h. Results showed that liraglutide treatment (50 nM) significantly reversed IL-1β-induced increase in GAG release from the extracellular matrix of the chondrocytes (*p* = 0.0227 compared with IL-1β alone) (Fig. 7c).

Overall, these results indicated that liraglutide may attenuate cartilage degradation in the long term via anti-catabolic effect, as demonstrated in vitro.

## Discussion

Repurposing clinically used drugs is an important strategy in drug discovery and could reduce cost and development timelines due to prior availability of safety and toxicity information^27,28^. The GLP-1/GLP-1R axis has been extensively studied in the context of diabetes. The GLP-1 analog liraglutide, a modified human GLP-1(7–37) with a longer half-life^29^, is administered systemically in patients with type II diabetes (commercial name Victoza^®^) and obesity (commercial name Saxenda^®^). Recently, liraglutide has gained increasing attention owing to its anti-inflammatory activities in age-related diseases^30^.

In the present study, it was observed that the liraglutide/GLP-1R axis acts in chondrocytes and macrophages to decrease inflammation and catabolism in vitro, which was confirmed by pain alleviation in vivo in mouse OA inflammatory pain model. Moreover, the expression of GLP-1R in OA patients indicated GLP-1R as a potential therapeutic target for OA treatment. The intra-articular injection of liraglutide for OA treatment could lead to drug repurposing.

GLP-1Rs are widely distributed in several cells, such as islet cells (especially β-cells of the pancreas), endothelial cells, neurons, adipocytes, keratinocytes, lymphocytes, hepatocytes, smooth muscle cells, myocytes, Brunner’s glands in the duodenum, and myenteric plexus neurons in the gut^31–34^. These pleiotropic expressions indicate that GLP-1 and its drugs can exert extra-pancreatic functions. Recently, the expression of the GLP-1 receptor has been reported in the knee joint of rat cartilage^35^ and in chondrocytes from mouse tracheal cartilage^36^, but has never been described in OA patients or in mouse articular cartilage and synovial membrane. However, the results of the present study showed that GLP-1 receptor was expressed in the cartilage and synovial membrane of both OA human and OA and non-OA mice.

Recently, Chen et al*.* showed that the activation of GLP-1R by liraglutide could protect chondrocytes against endoplasmic reticulum (ER) stress, apoptosis, and inflammation by decreasing the release of inflammatory mediators^35^ in a surgically induced rat model of OA knee. Additionally, Que et al*.* demonstrated that the anti-inflammatory effects of liraglutide act through the activation of the PKA/CREB pathway in a rat model of OA knee^37^. However, the potential analgesic effect of liraglutide is yet to be reported.

Pain, the major symptom of OA, is triggered by peripheral and central changes within the pain pathways. The mechanisms of pain in OA are complex and not yet well understood. It does not seem to be entirely driven by tissue damage, as highlighted by the discordance between pain and structural changes in OA joints^38^. Cartilage itself is not innervated, but nociceptors are present in surrounding tissues, such as the synovium and joint capsule, subchondral bone, periosteum, meniscus, and ligaments^39^. GLP-1R is expressed in dorsal root ganglion (DRG) neurons^40^ and could explain the potential direct effect on pain in OA since DRG neuron endings are present in the synovium^41^.

To explore the potential effect of liraglutide on OA-associated pain, MIA OA mice models were developed. This model has become a standard for modelling joint disruption in inflammatory OA in rodents. MIA injection to the knee joint leads to the progressive disruption of cartilage, which is associated with the development of pain-like behavior that mimics that of human OA. Thus, the MIA OA model is appropriate for studying the pharmacological effects of new drugs that can act on OA-related pain^42,43^. The findings of the present study indicated a dose-dependent analgesic effect of liraglutide in MIA OA mice model, with better results than dexamethasone (positive control treatment).

Local action seems to be paramount in sustaining an analgesic effect. In humans suffering from obesity, daily systemic injections of liraglutide did not ameliorate OA-related pain, probably because of poor access and hence poor local concentrations of liraglutide in the knee joint^44^.

To explain the analgesic effects of liraglutide, we hypothesized that its anti-inflammatory properties could play a central role. Recently, low-grade inflammation and innate immunity have been increasingly implicated in OA pathogenesis^13,45^. Synovial inflammation is associated with pain sensitization in patients with knee OA^46^, suggesting a role of pro-inflammatory mediators in this process^39^. Cytokines have been shown to sensitize knee joint nociceptors to mechanical stimulation in rats^47^. Moreover, inflammatory mediators can stimulate the distal terminals of DRG neurons, which in turn transmit sensory information to the central nervous system.

In vivo, we found that liraglutide improved the severity of synovitis in the MIA mouse model. Moreover, we conducted in vitro experiments on two murine cell types (chondrocytes and macrophages), reflecting two crucial cell types present in the knee joint. Immunohistochemical staining indicated the presence of GLP-1R at the membrane and cytoplasmic levels in both chondrocytes and macrophages. Additionally, inflammatory molecules produced by chondrocytes in response to IL-1β included PGE_2_ and cyclooxygenase (*Cox-2*). Moreover, IL-1β can induce the accumulation of reactive oxygen species (ROS), through the expression of *iNos*. Furthermore, the results of the present study showed that liraglutide induced a dose-dependent anti-inflammatory effects in murine primary chondrocytes. Recently, Chen et al*.* demonstrated the anti-apoptotic and anti-inflammatory effects of GLP-1R activation in rat chondrocytes. Regulation of PI3K/Akt/ER stress is closely involved in the protective effects of GLP-1R^35^.

Although fewer synovial macrophages are present in OA than in rheumatoid arthritis, they are crucial for the production of pro-inflammatory cytokines, such as IL-6 and TNF-α^11^. Previous studies have shown that selective depletion of synovial macrophages during experimental OA largely reduces cartilage damage and osteophyte formation, which are two major hallmarks of OA^48^. In LPS-stimulated macrophages, liraglutide significantly decreased *Il-6*, *Cox2*, and *Tnf-α* expression levels as well as PGE_2_, NO, and IL-6 concentration.

Furthermore, macrophage polarization may play a role in OA progression. Indeed, a higher ratio of M1/M2 was observed in synovial fluid from OA knee compared with normal knees, and the ratio was significantly correlated with the Kellgren–Lawrence grade^49^. However, it should be noted that macrophage phenotype plasticity in response to environmental conditions provides a broad spectrum of possibilities depending on their interactions^50^; thus, the classification of macrophages into the M1/M2 subtype is restrictive. In the present study, liraglutide promoted macrophage repolarization from M1 to M2 anti-inflammatory macrophage subtype in vitro. Thus, these results suggest that liraglutide could reduce, at least in part, the secretion of pro-inflammatory cytokines in OA synovium by stimulating macrophages and polarization toward an M2 anti-inflammatory phenotype, leading to a decrease in OA-induced joint destruction. However, these in vitro results on macrophage phenotype switch are still hypothetical and need to be tested in vivo. The effect of liraglutide on macrophage polarization has been verified in other tissues. Swada et al*.* recently revealed that liraglutide ameliorates the development of periodontitis, which was demonstrated by a decrease in periodontitis-induced inflammation, decrease in M1 macrophages in the gingiva, and alveolar bone loss with decreased osteoclast formation^51^. Moreover, Wan et al*.* demonstrated the effect of GLP-1 on macrophage activation, which contributed to M2 polarization and secretion of anti-inflammatory factors in RAW 264.7 cells^52^.

Previous studies have demonstrated that the effect of GLP-1 involve both GLP-1R-dependent and-independent pathways^53,54^. To further investigate the anti-inflammatory effects of liraglutide and determine if the GLP-1R signaling pathway is involved in its anti-inflammatory effects, exendin fragment 9–39, a competitive GLP-1R antagonist was added to the incubation. The results of the present study demonstrated that exendin 9–39 at 100 nM completely blocked the anti-inflammatory effect of 50 nM liraglutide treatment. These data demonstrated that GLP-1R is the primary target of liraglutide in chondrocytes and macrophages and that the anti-inflammatory effect of liraglutide was mediated by the GLP-1 receptor pathway in both chondrocytes and macrophages. However, to further validate these results, the relationship between GLP-1 content and GLP-1R expression in response to liraglutide should be examined in future studies.

Targeting cartilage breakdown by reducing catabolic enzyme activity is an important strategy for treating or modify the course of OA^55^. Liraglutide treatment in chondrocytes significantly reduced IL-1β-induced expression of *Adamts4*, *Adamts5*, *Mmp-3* and *Mmp-13* in a dose-dependent manner, leading to a decrease in MMP-3, MMP-13 and soluble GAG release by chondrocytes and macrophages. Taken together with its analgesic effects, these results indicated that liraglutide may attenuate cartilage degradation by inhibiting the expression of catabolic genes and protein in inflamed chondrocytes, as demonstrated in vitro. However, the findings of this study is limited by the small number of experiments performed, thus necessitating future studies to validate the findings of the present study. To validate this structural effect, it will be necessary to use an animal OA rodent model, such as the surgical destabilization of the medial meniscus (DMM), which is a more acceptable and suitable model to study the effects of liraglutide on cartilage degradation^56^.

Moreover, metabolic disturbances, such as obesity or diabetes mellitus, are associated with osteoarthritis, but data on the link between OA and lipid disturbances remain conflicting. A meta-analysis demonstrated an association between OA and dyslipidemia, indicating the role of metabolic disturbances in OA pathophysiology^57^. These results reinforce the concept of metabolic syndrome-associated OA phenotype. Liraglutide, like either approved GLP-1 drugs, has been shown to reduce weight, glucose and lipids in patients with Type 2 Diabetes Mellitus but also patients with overweight and obesity. GLP-1 signals through receptors primarily in the pancreas, gut and brain to increase insulin signalling and reduce food intake^58^. An extensive study of these metabolic changes, such as the effect of weight and glucose change on OA pain score, in response to liraglutide treatment is necessary. However, the present study is unique because it focused on the local effects of intra-articular injection of liraglutide and any potentially induce transient metabolic changes.

Disease-modifying osteoarthritis drugs (DMOADs) are a class of agents that target key tissues involved in OA, and according to FDA guidelines, they must prevent structural progression and improve symptoms^5,59^. Currently, no DMOADs have been approved for use, but a number of potential therapies are in clinical trials^60^. However, all drugs in development face the challenge of showing both analgesic and structural properties^61–64^. Owing to its analgesic, anti-inflammatory, and anti-catabolic activities, liraglutide constitutes a new potential DMOAD for clinical development.

Overall, the findings of the present study demonstrated that intra-articular stimulation of the liraglutide/GLP-1R axis has analgesic effects in vivo*,* which was probably due to the anti-inflammatory and anti-catabolic effects of liraglutide in vivo and in vitro, as demonstrated in vitro in macrophages and chondrocytes. Owing to its integrated mechanism of action on relevant cell types and the amelioration of OA symptoms, liraglutide could be a potential DMOAD for treating OA.

## Methods

### Chemicals and reagents

The GLP-1 analog, liraglutide, was purchased from Hybio Pharmaceuticals (Shenzhen, China) and Novo Nordisk (Bagsværd, Denmark). Dexamethasone was purchased from Mylan (Paris, France). Primary rabbit polyclonal immunoglobulin G (IgG) anti-GLP-1R antibody was purchased from Novus Biologicals (NBP1-97308, Centennial, Colo., USA). ImmPRESS^®^ horseradish peroxidase (HRP) goat anti-rabbit IgG polymer detection kit, BLOXALL^®^, VectaFluor^®^ kit with anti-rabbit IgG Dylight 488 antibody, and VECTASHIELD^®^ Antifade mounting medium (4′,6-diamidino-2-phenylindole—DAPI) were purchased from Vector Laboratories (Burlingame, CA, USA). The 3,3′-diaminobenzidine (DAB) substrate kit was purchased from Cell Signaling Technology (Leiden, Netherlands). IL-1β was obtained from PeproTech (Neuilly-sur-Seine, France). Collagenase D was purchased from Roche (Basel, Switzerland), Griess reagent system was purchased from Promega (Charbonnière-les-Bains, France), mouse PGE2, IL-6, MMP-3 enzyme linked immunosorbent assay (ELISA), and LDH assay kits were purchased from Abcam (Cambridge, United Kingdom). Mouse ELISA kit for MMP-13 was purchased from Cloud-Clone Corp. (Katy, TX, USA), and glycosaminoglycans (GAG) assay kit was purchased from Chondrex (Redmond, Washington, USA). All culture media and chemicals, including exendin 9–39, were purchased from Sigma-Aldrich (St. Louis, MO, USA) unless otherwise stated.

### Human samples

Human OA samples were isolated from the knees of six patients of different age (three women and three men; 68 to 83 years old for men, 71 to 83 years old for women) with varying osteoarthritis severity (Mankin score: 1.5–14 out of 14), undergoing joint arthroplasty at Assistance public—Hôpitaux de Paris (AP-HP) Saint-Antoine Hospital (Paris, France)^65^. For cartilage, 10 mm^2^ plugs of human OA cartilage and subchondral bone were obtained. Each plug included the entire cartilage layer and a piece of subchondral bone below the cartilage. Samples were fixed in 3.7% paraformaldehyde (PFA) at 4°C for 24 h and then decalcified in 500 mM ethylenediaminetetraacetic acid (EDTA) (pH 7.4, VWR, Pennsylvania, USA)^66^. Synovial membrane samples were dissected from adjacent adipose tissue and fixed in 3.7% PFA for 24 h. Cartilage or synovial membrane samples were embedded in paraffin, and 5 µm sagittal sections were obtained using a Polycut E microtome.

Patients’ data were anonymized, and informed consent for the use of tissue was obtained from each patient before surgery. Experiments using human samples were approved by French Institutional Review Board (Comité de Protection des Personnes, Paris Ile-de-France 5 and Commission Nationale de l’Informatique et des Libertés). All experiments were performed in accordance with relevant guidelines and regulations.

### Immunohistochemical staining

The presence of GLP-1R in OA human knee joint sections harvested following arthroplasty and non-OA mouse knee joint sections harvested following a mouse study was evaluated using immunohistochemical staining. The sections were deparaffinized and rehydrated. Following incubation at 60°C overnight in 10 mM sodium citrate buffer (pH 6.0) for antigen unmasking, BLOXALL^®^ blocking solution was added to inactivate endogenous peroxidase (10 min at room temperature (RT)). Slides were then blocked with Tris-buffered saline (TBS) + 0.05% Tween^®^ 20 with 5% non-fat milk and 3% BSA, followed by 2.5% normal goat serum. Tissue sections were incubated with human anti-GLP-1R primary antibody (0.02 mg/mL) at 4°C overnight and then with secondary goat ImmPRESS^®^ HRP anti-rabbit IgG antibody at room temperature for 30 min. The staining was revealed using DAB chromogen solution. Hematoxylin was used to counterstain nuclei (20 s). Images were captured using a Zeiss Axioplan 2 imaging microscope and analyzed using ImageJ software.

### Immunofluorescence staining

Primary murine chondrocytes and RAW 264.7 murine macrophage cell line were fixed with 3.7% PFA for 30 min at RT, permeabilized with TBS + 0.05% Tween^®^ 20 buffer for 30 min at RT, and blocked with 2.5% normal horse serum for 20 min at RT. Next, cells were incubated with 0.01 mg/mL primary antibody and then with secondary VectaFluor^®^ anti-rabbit IgG Dylight 488 antibody for 30 min at RT. VECTASHIELD^®^ Antifade mounting medium with DAPI was used for microscopic observation. Images were captured using a spinning disk confocal microscope (CSU-W1, Nikon) and analyzed using ImageJ software.

### Sodium monoiodoacetate (MIA) model of OA in mice

Animal handling was performed according to the guidelines of the Federation of European Laboratory Animal Science Associations (FELASA). All experiments involving animals were performed following approval from the Ethics Committee for Animal Experimentation of Nord-Pas de Calais Region (CEEA75, APAFIS#2019012113002115), and in compliance with the Animal Research: Reporting of In Vivo Experiments (ARRIVE) guidelines.

Wild-type male C57Bl/6 mice (12 weeks of age) were purchased from Janvier Labs (Le Genest-Saint-Isle, France). For the short-term MIA model, 120 animals were divided into seven groups: saline/vehicle group (n = 19 mice), MIA/vehicle group (n = 18), all MIA/liraglutide groups (n = 17), and dexamethasone group (n = 15). For the long-term MIA model, 49 animals were divided into four groups: saline/vehicle (n = 9 mice), MIA/vehicle groups (n = 10), MIA/liraglutide (n = 10), or dexamethasone (n = 10).

Mice (20–35 g) were maintained in plastic ventilated cages in a pathogen-free environment (12 h/12 h light/dark cycle, 21–24°C) at Institut Pasteur de Lille, and had ad libitum access to water and food. Mice were anesthetized with isoflurane (2–5% in O_2_) before intra-articular (IA) injection. Osteoarthritis was induced in the right knee joint through a single IA injection of MIA (0.75 mg/5 µL sterile 0.9% saline, pH 7.4) on day 1. The MIA dose was selected based on a previously published study^42^. Mice in the control group were injected with an equivalent volume of saline. Following MIA induction, mice were allocated into treatment groups based on the results of von Frey test, which was performed on day 2 for short-term MIA model or day 7 for long-term MIA model. Mice were tested to quantify nociceptive threshold on days 7 and 10 in the short-term model and on days 14, 21, and 28 after injection of MIA or saline in the long-term model. Animals were sacrificed on day 11 (short-term model) or day 29 (long-term model).

For the MIA short-term study, mice received a single IA injection of liraglutide (1, 5, 10, or 20 µg) or empty vehicle (5 µL) into the right knee on day 3. In the long-term MIA study, mice were administered 20 µg liraglutide or vehicle control once a week for 3 weeks on day 8, 15, and 22. Dexamethasone (20 µg) was used in these two experiments as an anti-inflammatory positive control following the same regimen (1 injection at day 3 in the short-term and 3 injections once a week from day 8 in the long-term MIA model). All measurements were performed by two observers blinded to the treatment. Mice were observed throughout the duration of each experiment for signs of mortality and morbidity, and overall appearance (activity, general response to handling, touch, ruffled fur). Mice were weighed twice per week.

### Pain assessment

Joint nociception was evaluated using the von Frey test (tactile allodynia). Mice were placed individually in transparent plexiglass chambers and acclimated for 15 min. Paw withdrawal threshold was calculated from the average of two trials, using Semmes–Weinstein von Frey monofilaments with bending forces of 0.008, 0.02, 0.04, 0.07, 0.16, 0.4, 0.6, and 1.0 g) applied in ascending order to the plantar surface. The lowest force from the test that produced a response was considered the withdrawal threshold. All procedures and testing were performed in a blinded manner.

### Histological analysis

After the short-term MIA study, mice were euthanized on day 11, and the entire knee joints were removed and fixed in 4% PFA at 4°C for 72 h and then decalcified with 14% EDTA solution (pH 7.4). After dehydration and embedding in paraffin, the tissues were cut into 5 µm thick sections using a Polycut E microtome and mounted on slides. Tissue morphology was evaluated by hematoxylin and eosin staining (H&E) to assess chronic synovitis according to the Krenn score. The scores were blindly assigned by two different examiners.

### Cell culture and treatments

The murine macrophage cell line RAW 264.7 (TIB-71, American Type Culture Collection, Manassas, VA, USA) was cultured at 37°C and under 5% CO_2_ condition in Dulbecco’s modified Eagle’s medium (DMEM) containing 4.5 g/L glucose, 10% fetal bovine serum (FBS), and 1% penicillin–streptomycin (P/S) mixture. RAW 264.7 cells were plated and treated in starving medium (DMEM containing 1% P/S). The macrophage cell line was used before passage 20 for all the experiments.

Murine primary chondrocytes were isolated from the knee articular cartilage of 6 days-old C57Bl/6 mice as previously described^67^. Cell morphology examination, Alcian blue and Safranin O staining, and qRT-PCR for chondrocyte markers (*Sox9*, *Col2a1*, and *Acan*) confirmed that the cells extracted from the heads, femoral condyles, and tibial plates of mice were differentiated chondrocytes. Chondrocytes were cultured 7 days before starvation (with 0.1% bovine serum albumin (BSA)) and treatments. Chondrocytes were used at passage 0 for all the experiments.

Murine primary chondrocytes or RAW 264.7 cells were stimulated with 2 ng/mL of IL-1β or 100 ng/mL of lipopolysaccharide (LPS), respectively, and co-treated with ascending concentrations of liraglutide (6.6, 13.3, 26.6, 53.1, 106.3, 212.5, 425, 850 nM, 1.7 and 3.4 µM) at 37°C + 5% CO_2_ for 24 h. Exendin fragment 9–39, a competitive antagonist of GLP-1R, was used at concentrations of 25 nM, 50 nM, and 100 nM. For long-term in vitro study, murine primary chondrocytes were stimulated with 2 ng/mL IL-1β and treated with 50 nM of liraglutide at 37°C and 5% CO_2_ for 72 h.

### Quantification of inflammatory and degradation marker

Total mouse PGE_2_, IL-6, MMP-3, and MMP-13 concentration of primary murine chondrocytes (p0) or murine macrophage cell line (p + 9) after 24 h of treatment was assayed in cell-free supernatants using an ELISA kit, according to the manufacturer’s instructions. The nitrite content of primary murine chondrocytes (p0) and murine macrophage cell line (p + 9) after 24 h of treatment was determined by the Griess method^68^ using a Griess Reagent System kit according to the manufacturer’s instructions. Glycosaminoglycans concentration of primary murine chondrocytes (p0) after 24 or 72 h of treatment was measured using GAG Assay kit, according to the manufacturer’s instructions. Lactate dehydrogenase assay (LDH) was performed to confirm the non-cytotoxicity of liraglutide treatment on both cell types. No enzymatic treatment was performed prior to the protein assay. Concentrations were analyzed using a spectrophotometer and determined by comparison against a standard curve.

### Real-time quantitative PCR

Total ribonucleic acid (RNA) was extracted from murine chondrocytes or macrophages using the ReliaPrep™ RNA Cell Miniprep System (Promega, Charbonnière-les-Bains, France). Thereafter, 1 µg of total RNA was reverse-transcribed to cDNA using the Omniscript^®^ RT kit (Qiagen, Courtaboeuf, France). To detect messenger RNA (mRNA) expression levels of GLP-1R downstream target genes, quantitative reverse transcription real-time polymerase chain reaction (RT-qPCR) was performed using GoTaq^®^ qPCR Master mix. The relative expression of target genes was calculated and normalized to that of hypoxanthine phosphoribosyltransferase 1 (HPRT) using the Delta C(T) method. The sequences of primers used are listed in Supplementary Table 1.

### Statistical analysis and IC_50_/EC_50_ determination

For all studies, groups were compared to control groups (saline or vehicle). A total of 15–19 mice per group and 9–10 mice per group were used for short-term and long-term studies, respectively. The choice of the number of experiments was established by power analysis tests and previously published work^42,69^. The small number of experiments used for each part of the work can be considered a limitation. All quantitative data were presented as mean ± standard error of mean (SEM). GraphPad software (version 9.0) was used to perform statistical analysis. Non-parametric Mann–Whitney (sequential strategy) or two-way ANOVA was used to analyze significant differences between groups, and statistical significance was set at *p* < 0.05. The inhibition percentages were analyzed using GraphPad Prism 9.0 and the half maximal inhibitory concentration (IC_50_) or half maximal effective concentration (EC_50_) values were determined.

## Supplementary Information

Supplementary Information.
