# Supplementary material for: Liraglutide, a glucagon-like peptide 1 receptor agonist, exerts analgesic, anti-inflammatory and anti-degradative actions in osteoarthritis
Source: Sci Rep. 2022 Jan 28;12:1567. doi: 10.1038/s41598-022-05323-7 (PMC8799666; doi:10.1038/s41598-022-05323-7)
Supplement: Supplementary file 1 — Supplementary Information. [file 41598_2022_5323_MOESM1_ESM.docx]

**Supplemental Materials**

**Supplemental Figures**


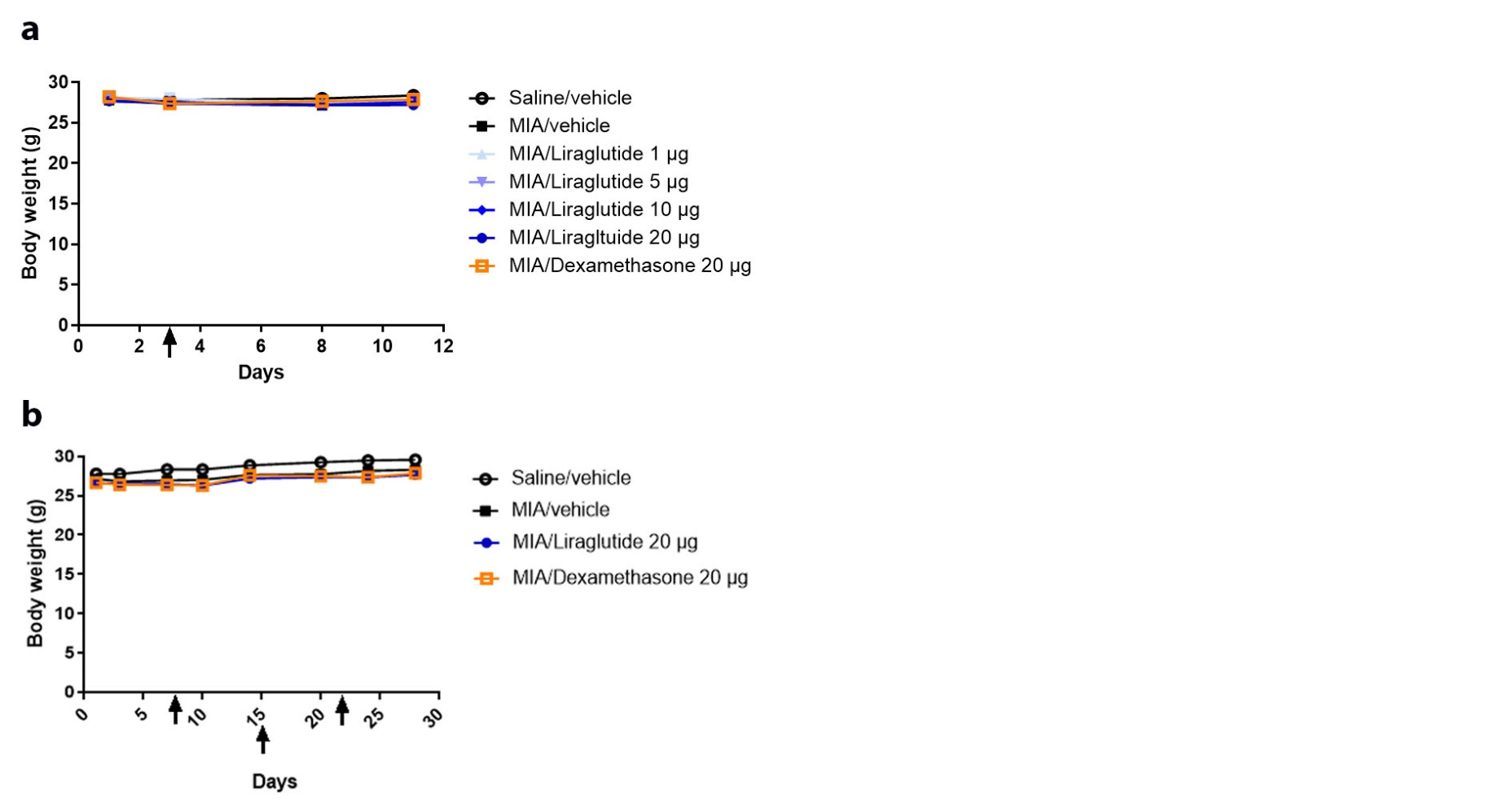


**Fig. supp 1: Liraglutide did not significantly affect the body weight of MIA mice model of OA.**

Mice knee joints were IA injected with 0.75 mg of MIA or saline on day 1. For short-term study (a), at day 3 (two days after OA induction), treatments (liraglutide, dexamethasone, or vehicle) were IA injected. For the long-term study (b), IA treatments were administered on day 8, 15, and 22. (a) Mean body weight of mice in the treatment groups on days 1, 3, 8, and 11 (n = 15-19 per group). (b) Mean body weight of mice in the indicated treatment groups on days 1, 3, 7, 10, 14, 20, 24, and 28 (n = 9-10 per group). Arrows indicate the treatment with IA administration. Statistical analysis: Mean ± SEM, two-way ANOVA test, no statistical differences between groups.


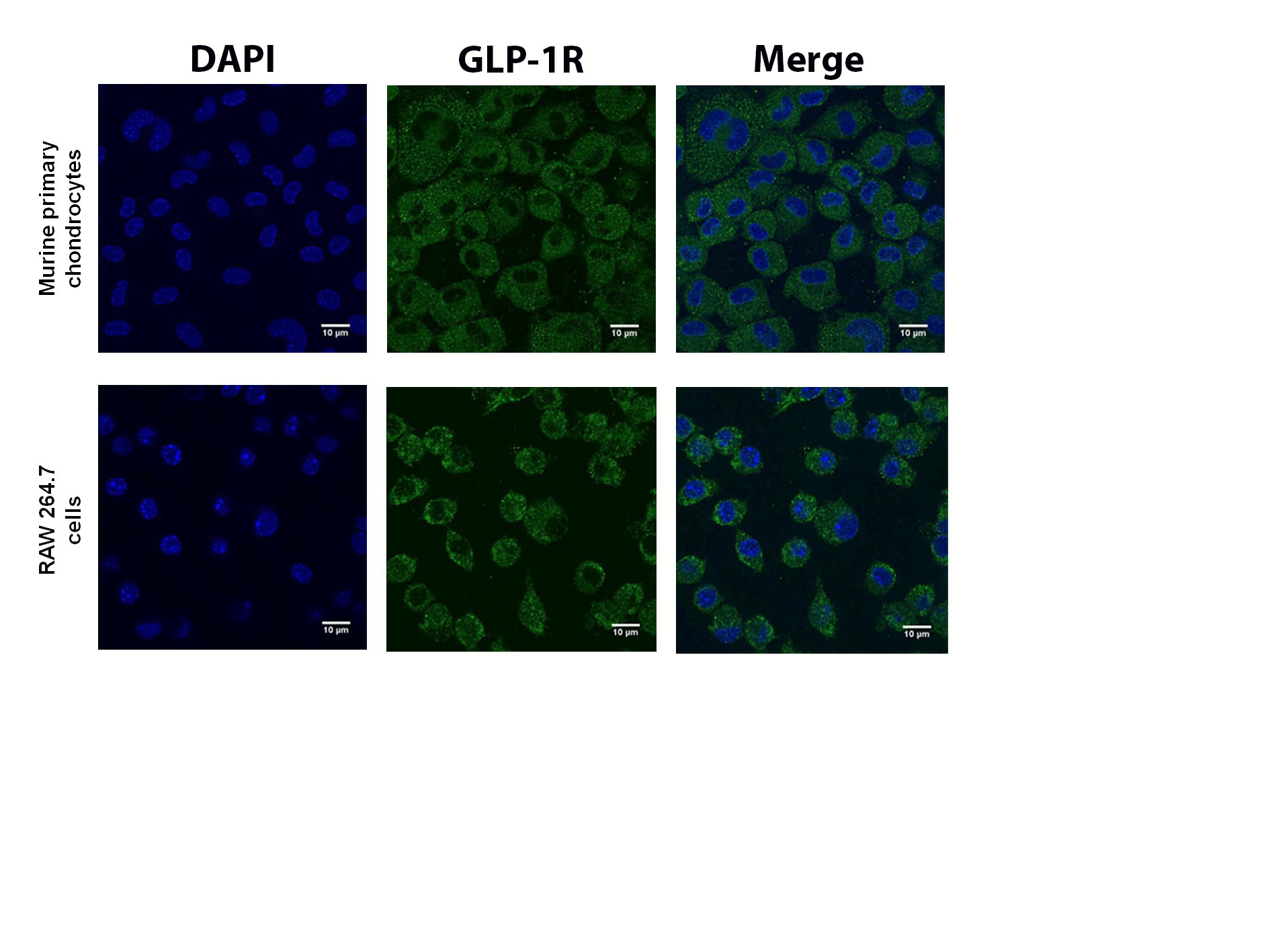


**Fig. supp 2: Expression of GLP-1 receptor in murine chondrocytes and macrophages.**

Immunofluorescence staining for GLP-1R was performed on murine primary chondrocytes and murine macrophage RAW 264.7 cells. The control was performed without incubation with primary antibody. Representative field of immunofluorescence staining of GLP-1R (green) and nuclei labeled with DAPI (blue) (scale bar = 10 µm).

**Supplementary Table 1: List of primers used in RT-qPCR for inflammation and catabolic genes.**

| **Genes** | **Forward primer** | **Reverse primer** |
| --- | --- | --- |
| *INos* | AAG-GTG-AGC-TGA-ACG-AGG-AG | GGA-GCC-TTT-AGA-CCT-CAA-CAG-A |
| *Cox2* | GCA-TTC-TTT-GCC-CAG-CAC-TT | AGA-CCA-GGC-ACC-GAC-CAA-AGA |
| *Tnf-α* | CAA-GGA-CTC-AAA-TGG-GCT-TTC-CG | TCT-GTC-AGG-AAG-GCT-GTG-CAT-TG |
| *Il-6* | GTC-ACA-GAA-GGA-GTG-GCT-A | AGA-GAA-CAA-CAT-AAG-TCA-GAT-ACC |
| *Mcp-1* | CAT-CCA-CGT-GTT-GGC-TCA | GAT-CAT-CTT-GCT-GGT-GAA-TGA-GT |
| *Cd38* | AAG-ATG-TTC-ACC-CTG-GAG-GA | ACT-CCA-ATG-TGG-GCA-AGA-GA |
| *Erg-2* | CTA-CCC-GGT-GGA-AGA-CCT-C | AAT-GTT-GAT-CAT-GCC-ATC-TCC |
| *Adamts4* | GGA-GGC-AGT-GAT-GTC-TTG-GTG-AAT | TCA-TCC-CTT-ACT-CCC-TGA-AGG-CA |
| *Adamts5* | ACT-CTT-GGC-CCC-ACT-GTA-TAA-GTC | TGT-ACG-GTC-CCA-TGT-TTC-AGT-G |
| *Mmp-3* | TGA-AAA-TGA-AGG-GTC-TTC-CGG | GCA-GAA-GCT-CCA-TAC-CAG-CA |
| *Mmp-13* | GAT-GGC-ACT-GCT-GAC-ATC-AT | TGT-AGC-CTT-TGG-AAC-TGC-TT |
